# Towards a New Euro Crisis?

**DOI:** 10.1007/s10272-022-1078-x

**Published:** 2022-10-10

**Authors:** Paul De Grauwe

**Affiliations:** European Institute, London School of Economics and Political Science, Houghton Street, WC2A 2AE London, UK

**Keywords:** E52, F33

## Abstract

There is thus a fundamental credibility issue about the willingness of the ECB to be a lender of last resort in the government bond markets. This will continue to make the eurozone a fragile construction. As a result, the possibility of a future euro crisis cannot be excluded.

Ten years ago, the eurozone experienced an existential crisis. During 2012, it became a popular sport to forecast the date when the system would crash and the euro would disappear. In those days when the sovereign debt crisis hit the eurozone, we learned that the system is fragile and that relatively small shocks could lead to its disintegration.

In this article, I take up the fragility problem of the eurozone. I discuss first the origin of this fragility. Then I look at the history of this fragility and analyse what has been done to the governance of the eurozone over the years to reduce its fragility and make it more sustainable. I conclude with the question: Have we done enough to strengthen the eurozone so that we can now be sure it is prepared to face future crises, in particular the inflation crisis that we are confronting today?

## Defining fragility

The eurozone is a fragile construction. Governments of the member countries of a monetary union issue bonds that they promise to convert at maturity in a currency, the euro, over which they have no control. It is as if each of these governments issues debt in a foreign currency. Like the Argentinian government when it issues bonds in dollars, it is a currency that this government does not control (Eichengreen et al., 2005).

As a result, the governments of a monetary union cannot give a 100% guarantee to the bondholders that they will have the necessary liquidity to pay them out at maturity. The risk that governments can run out of cash in a monetary union creates the potential for self-fulfilling liquidity crises: investors who are afraid that the government may run out of cash, panic and massively sell that government's bonds, thereby precipitating the liquidity crisis that they were afraid of. Such a crisis may force the government to default on its debt (De Grauwe, 2011; Beirne and Fratscher, 2012; De Grauwe and Ji, 2013; Aizenman et al., 2013; Montfort and Renne, 2013).

**Paul De Grauwe**, London School of Economics and Political Science, UK.

This problem does not exist in stand-alone countries where governments issue debt in their own currency. Investors know that these governments are backed by a central bank that is ready (or can be forced) to provide all the cash that is necessary to that government in times of crises. Thus, investors cannot force default on a stand-alone country's government. They can force default of national governments in a monetary union; an extraordinary implication of a monetary union that was overlooked when the eurozone was created. This also implies that the balance of power shifted in favour of financial markets against the sovereigns when countries joined the monetary union. A paradoxical situation because we thought that being in a union would make the member countries stronger. Exactly the opposite occurred: By entering the monetary union, the governments of the member countries were weakened while the power of financial markets over these governments increased.

## The history of the eurozone's fragility

The best measures of the fragility of the eurozone are the "spreads" in the government bond markets. These are the differences in the 10-year government bond yields of member countries with the 10-year government bond yield of Germany. The underlying assumption is that the German government bond yield is risk-free. Therefore, any positive difference between the yield of the bond of a particular government and that risk-free rate expresses the risk investors attach to holding the bond of that government. Let us look at the spreads from 2000 and 2022 (Figure 1).

The period 2000-2022 is divided into three sub-periods: the pre-crisis, the crisis and the post-crisis periods. These three sub-periods can instruct us about how the fragility of the eurozone evolved over time.

### Pre-crisis period

During this period, the spreads were virtually zero. This implies that government bonds in the eurozone countries were seen as (almost) perfect substitutes. Thus, investors considered the risk involved in holding, say, a Greek government bond to be the same as the risk in holding a German bond. One can say that this was the honeymoon

Figure 1

**Ten-year government bond spreads, selected eurozone countries, 2000-2021**

in %

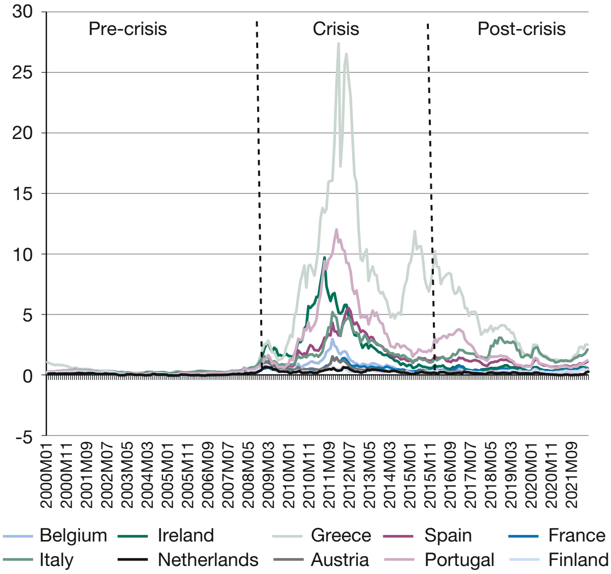

Source: Eurostat.

period of the eurozone. Everything looked beautiful, no clouds in the sky. A remarkable situation during which investors and policymakers lived in a fantasy world.

## Crisis period

The financial crisis of 2008 completely changed the risk perceptions in the government bond markets. The governments of those countries hit most by the financial crisis saw their budgetary and debt situation deteriorate quickly. As the national government bond markets lacked a backstop, i.e. a central bank willing to provide liquidity in the government bond markets in times of crisis, the self-fulfilling liquidity crises described earlier were set in motion. These self-fulfilling crises had further dramatic effects. They led some countries to be pushed into "bad equilibria" and others into "good equilibria" (see De Grauwe and Ji, 2013).

*Good and bad equilibria*

The governments of the high-risk bond markets were pushed into a bad equilibrium: the need to find liquidity forced these governments to raise taxes and to reduce spending. These forced austerity programmes in turn made the recession worse and intensified the debt problem of these governments. The governments of the low-risk countries had plenty of liquidity and were spared the need to install severe austerity. All this led to an existential crisis of the eurozone.

It also led to an unsustainable political situation where the creditor countries that had received massive inflows of capital dictated the austerity programmes to the high-risk countries, which suffered twice. Once because the harsh austerity programmes led to unnecessary suffering for millions of people. And a second time because these programmes felt like a foreign intrusion and a humiliation. It became clear that a repetition of this economic and political crisis in the eurozone would lead to its demise.

## Post-crisis period

It took the ECB until September 2012 to accept its responsibility: The ECB then announced that it was ready to provide unlimited liquidity support in the government bond markets. This so-called Outright Monetary Transactions (OMT) programme started a process of normalisation during which yields gradually converged again. This convergence was sometimes bumpy, as during the second Greek crisis in 2015. It ultimately led to an almost complete convergence of the yields at the end of 2019.

By promising unlimited purchases of government bonds during a liquidity crisis, the ECB took the fear factor out of the market. Suddenly, Greek, Spanish and Italian bonds, whose prices had collapsed as a result of fear of liquidity shortages, appeared to be cheap for private investors. They massively returned to these bond markets, bought the bonds and raised their prices. The spreads collapsed quickly (see Figure 1).

This recovery showed the importance of having a lender of last resort in the government bond markets, i.e. a central bank willing to provide unlimited amounts of liquidity in the government bond markets. In doing so, the ECB actually mimicked what central banks in stand-alone countries do. It also saved the eurozone.

Then came the coronavirus pandemic in 2020. There was a risk that the huge shock that hit the eurozone countries would trigger a new sovereign debt crisis, especially since the high-risk countries in the periphery also appeared to have suffered significantly larger negative effects on their GDP than low-risk countries (see Figure 2).

The sovereign debt crisis did not happen. In fact, apart from an early hiccup in the yields of Italy and Greece, the yields continued to converge further (see Figure 3) so that at the end of September 2021 the spreads were even smaller than before the onset of the pandemic (see Candelona et al., 2021).

Figure 2
**GDP growth during 2020**
in %

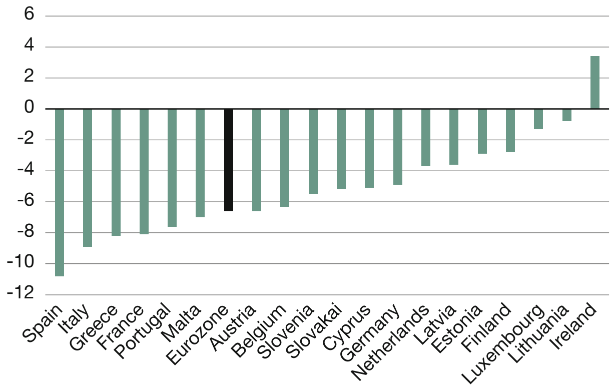

Source: Eurostat.

Figure 3
**Ten-year government bond spreads, selected eurozone countries, 2019-2022**
in %

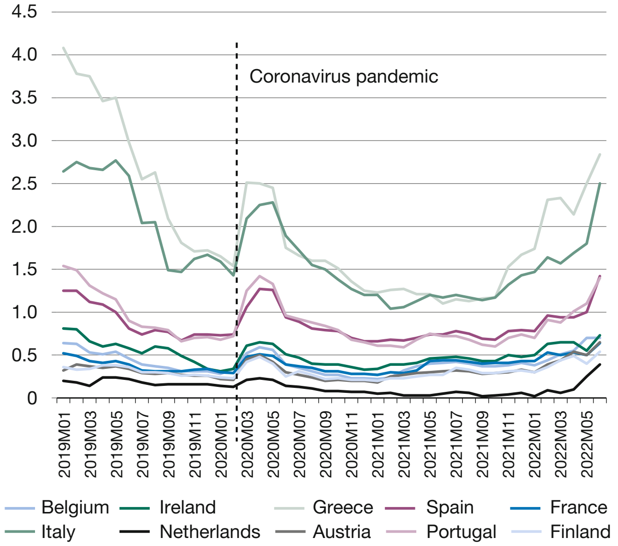

Source: Eurostat.

## Why did the pandemic not lead to a crisis?

Despite the large differences in the economic impacts of the pandemic, this shock did not lead to a new sovereign debt crisis. How did this remarkable result come about? My answer is that the new governance of the eurozone that emerged after the sovereign debt crisis of 2010-12 allowed European policymakers to use new instruments of stabilisation. As a result, the fragility of the eurozone was significantly reduced, thereby making it possible to avoid self-fulfilling crises in the government bond markets. The new instruments that achieved this result were both monetary and fiscal.

*Monetary instrument*

The ECB's decision to launch the OMT programme in 2012 created the single most important monetary instrument in the stabilisation of government bond markets in the eurozone at that time. This led to the expectation during the pandemic that the ECB would be ready to intervene in times of crises, which pacified markets. The expectation was reinforced by the ECB's announcement of its Pandemic Emergency Purchase Programme (PEPP) in 2020. This was a programme of large-scale government bond purchases by the ECB. The innovation of this programme was the absence of conditionality. While the OMT programme was linked to an austerity programme by governments receiving aid, the PEPP was stripped of any such austerity requirements. This was a remarkable intellectual reversal of the ECB policy towards support of the government bond markets. It was also the correct reversal because the ECB should only intervene in the government bond markets to solve a liquidity crisis triggered by the fear and panic of investors. There is no reason that governments receiving such support should be subjected to the condition that they impose austerity. It was refreshing to see that the ECB learned from past mistakes.

As a result of this new monetary governance, the spreads quickly declined again so that at the end of 2020, they were lower than they were at the end of 2019.

*Fiscal instrument*

A second major policy innovation was a fiscal one. After much controversy, the European leaders decided in July 2020 to set up a recovery plan amounting to €807 billion. The NextGeneration EU (NGEU) plan was funded by the issue of common bonds. Half of the proceeds of this bond issue were to be used as transfers (not loans) to those countries most hit by the pandemic. This was an important step towards a budgetary union in which a central authority obtains the power to issue debt that is guaranteed jointly by all member countries. It was the first issue of Eurobonds.

This common spending programme financed by the issue of Eurobonds helped instil confidence in the future of the eurozone. It signalled that the path of the monetary union would be one involving further steps towards a budgetary union. This was the second reason why the COVID-19 shock did not lead to a sovereign debt crisis.

## Today's challenge: Will the surge in inflation lead to a new surge in fragility?

The surge in inflation creates two dilemmas for the ECB. The first dilemma is the traditional one that every central bank, including the ECB, faces after a supply shock. This dilemma can be described as follows: During the past year, major supply shocks occurred – energy and commodity prices increased dramatically, the cost of production increased and inflation surged in most countries as a result. It also led to losses of purchasing power so that production was negatively affected. This is often called stagflation. Stagflation is at the core of the dilemma faced by the central bank. If the latter wants to fight inflation, it will have to raise the interest rate. But this will have a negative effect on production. It may even lead to a recession. If, on the other hand, the central bank wants to avoid a recession, it cannot increase the interest rate too much but then inflation may not easily go down and may become a permanent feature. This leads to a very uncomfortable dilemma for the central bank. Whatever it chooses, the outcome will be painful: Fighting inflation may produce a recession, but fighting a recession may make inflation permanent. Most central bankers have elevated reducing inflation as their primary objective so that it looks likely that they are willing to risk a recession to fight inflation.

The second dilemma is the one that the ECB faces as a central banker of a monetary union (in addition to the one previously mentioned). This second dilemma can be described as follows: When the ECB raises the interest rate, it has very different effects on the long-term bond rates of the different member countries. Every one percentage point increase of the long-term rate of Germany leads to an amplified effect on the long-term rate in high-risk countries. As can be seen from Figure 3, the spreads of Italy and Greece that were close to 1% at the start of 2022 have now moved in the 2.5%-3% range. This is confirmed in Figure 4. Further increases in the interest rate triggered by the ECB's desire to fight inflation could lead to an explosion of the spreads and risk creating a new sovereign debt crisis.

Thus, the second dilemma the ECB faces is the choice between fighting inflation at the risk of creating financial instability in the eurozone, or fighting financial instability at the risk of losing the battle against inflation – an equally uncomfortable dilemma as the first one.

There are two ways out of this dilemma. Both, however, create new discomforts. The first way out consists of a

Figure 4

**Change in the yield of the 10-year government bond rates per 1% change of the German bond rate, January to June 2022**

in %

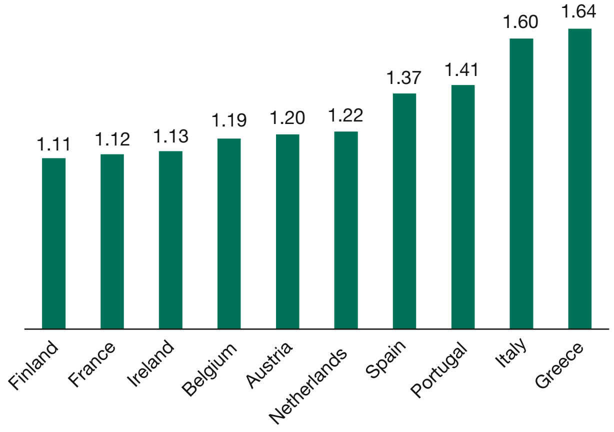

Source: Eurostat.

commitment by the ECB to provide an unlimited amount of liquidity to countries experiencing liquidity crises. In fact, in July 2022 the ECB announced a new programme, the Transmission Protection Instrument (TPI), which does exactly that: It provides liquidity to governments experiencing liquidity crises.[1] However, this will create additional liquidity in the system, which will interfere with the central bank's desire to fight inflation. The ECB will therefore have to withdraw liquidity from the system by selling government bonds from low-risk countries (Germany, the Netherlands, Finland). As a result, the ECB will increasingly accumulate high-risk government bonds at the expense of low-risk government bonds. This may create political problems when countries like Germany and the Netherlands resist.

There is a second potential way out of this dilemma. It consists of allowing inflation to increase above the self-imposed target of 2%. Several academic economists have argued that 2% is too low a target and that a target range of 3% to 4% would be more appropriate (see Blanchard, 2010; Ball, 2014; De Grauwe and Ji, 2019), mainly because it would make it less likely that central banks get trapped in the zero-lower-bound syndrome that has made monetary policies so ineffective for so long.

Raising the inflation target would not eliminate the dilemma but it would make it less constraining, thereby reduc-

1 For more information on TPI see https://www.ecb.europa.eu/press/pr/date/2022/html/ecb.pr220721~973e6e7273.en.html.

ing the probability of future crises. This way out from the dilemma, however, would trigger uncomfortable political problems similar to the previous one.

## Prospects for the future

Since the sovereign debt crisis of 2010-12, a new governance of the eurozone has emerged. This has made it possible for the eurozone to withstand the major economic disruptions brought about by the pandemic. But a new risk has emerged: inflation.

Will the need to fight inflation with higher interest rates again reveal the fragility of the eurozone? This leads to the question of whether the eurozone has now matured and permanently eliminated its fragile nature.

There is a fundamental contrast between the eurozone and stand-alone countries, i.e. countries with their own central bank. In a stand-alone country, the central bank faces one sovereign, which always prevails in times of crisis. There can be no doubt that in a stand-alone country the central bank will have to provide liquidity when the government faces a liquidity crisis.

In the eurozone, things are very different. The ECB faces 19 sovereigns (20 as of 1 January 2023 with the entry of Croatia), none of which has authority over the ECB. None of these governments can force the ECB to provide liquidity in times of crisis. The decision to provide liquidity support is at the discretion of the central bank. This creates uncertainty about future liquidity support in a monetary union, an uncertainty that is absent in stand-alone countries.

One can have reasonable doubts about the question of whether the ECB will always be ready in the future to provide liquidity support to the sovereigns. Who will be at the helm of the bank in the future? Will the Governing Council that consists mainly of national central bankers always be receptive to the demand of one member country's government for support?

One cannot be sure about this; it stands in stark contrast with the certainty we have that if, for example, the British government were to experience a liquidity shortage, the Bank of England will always step in.

There is thus a fundamental credibility issue about the willingness of the ECB to be a lender of last resort in the government bond markets. This will continue to make the eurozone a fragile construction. As a result, the possibility of a future euro crisis cannot be excluded.
